# Innovative bedding materials for compost bedded pack barns: enhancing dairy cow welfare and sustainable dairy farming

**DOI:** 10.1007/s10668-024-05244-7

**Published:** 2024-07-17

**Authors:** Daniela T. Eberl, Marshall J. Smith, Oliver J. Megram, Megan M. Mayhew, Debra Willoughby, Samuel J. White, Philippe B. Wilson

**Affiliations:** 1 Medical Technologies Innovation Facility, Nottingham Trent University, Clifton Campus, College Drive, Nottingham, NG11 8NS UK; 2 School of Science and Technology, Nottingham Trent University, Clifton Campus, College Drive, Nottingham, NG11 8NS UK; 3 Grange Farm, Butt Lane, Normanton on Soar, Loughborough, Leicestershire, LE12 5EE UK; 4 York St John University, Lord Mayor’s Walk, York, YO31 7EX UK

**Keywords:** Animal welfare, Carbon materials, Circular economy, Dairy, Livestock bedding, Sustainability

## Abstract

Compost bedded pack (CBP) barns are an innovative housing system that improves the comfort and welfare of dairy cows, compared to cubicle style housing or free stalls with artificial surfaces, such as rubber or concrete. This type of bedding system also has the potential to improve lameness scores, overall health, welfare, and productivity of dairy cows. In CBP barns, carbon materials or organic materials are composted in the barn while being used as bedding for livestock. The animals pass manure on these surfaces providing the nitrogen, microorganisms, and moisture necessary for the composting process. Historically, dry sawdust originating from mills, furniture and pallets have been used as a substrate for compost. However, due to these materials becoming increasingly expensive and hard to source, other materials have been trailed as potential substitutes. Furthermore, there is an increasing interest in making dairy production more environmentally friendly by reducing carbon footprint. This review summarises and highlights appropriate alternative materials that, subject to their management, can be successfully used in the CBP barn system. This will act as an aid for farmers and decision makers when choosing materials to be incorporated in CBP barns. Using alternative materials to sawdust, wood chips and wood shavings, which are the current industry standard, will contribute to a more circular economy and sustainable dairy production, while simultaneously contributing to sustainable development goals, and improved animal health and welfare.

## Introduction

Compost-bedded pack barns (CBP) are an innovative loose housing system that enhances comfort for livestock, such as dairy cows. In this system, the animals are not confined to individual stalls or housed on hard or artificial surfaces such as rubber mats or concrete. Instead, cows are housed in large barns with an open bedded pack area that offers increased cushioning and in which they can freely roam and rest (Bewley et al., 2017), which has a positive impact on animal welfare (Zhang et al., 2019). In CBP barns, the surface area required per animal is greater than in traditional housing systems. Different organic materials are combined with the urine and manure, and the mixture is stirred and aerated between one and three times a day. In this type of system, the materials that are mixed with the manure and urine, absorb moisture, and go through a composting process while simultaneously being used as a bedding substrate. Historically, dry sawdust originating from mills, furniture and pallets have been used as a substrate for the compost. However, due to these materials becoming increasingly expensive and hard to source, other materials have been trialled as potential substitutes (Shane et al., 2010).

As the name indicates, the CBP barn system relies on composting carbon materials along with cow manure within the barn. Composting is the process through which organic compounds—usually organic waste materials—are turned into compost, a material which is rich in nutrients to fertilise and condition land. The composting process is made up of four main stages: (1) mesophilic, (2) thermophilic and hygienisation, (3) second mesophilic phase, and (4) maturing phase (Meena et al., 2021).

The first phase begins within hours of creating the compost mix. Microbial activity (fungi, bacteria and actinobacteria) causes the temperature of the organic matter to rise to between 25 and 40 °C. Sugars and other soluble compounds such as proteins start to decompose and induce a drop in pH due to the production of organic acids. This phase typically lasts between two and eight days (Insam & Bertoldi, 2007; Meena et al., 2021). After the first phase is completed, thermophilic microorganisms, which thrive at higher temperatures (35 to 65 °C), largely replace the mesophilic microorganisms from phase one. In phase two, lignin and cellulose are decomposed, and a rise in pH occurs due to the conversion of ammonium to ammonia. Common harmful microorganisms, such as *Salmonella spp.*, *Escherichia coli* as well as parasite cysts are killed in this phase. This process contributes to the hygienisation of the compost material and is the result of high temperatures as well as the production of antibiotics by actinobacteria (Meena et al., 2021). In some cases, the temperature in phase two can reach 80 °C due to abiotic processes which may involve enzymes from actinobacteria. During the third phase, the supply of high-energy compounds, required for the survival of thermophilic microorganisms, becomes depleted and the temperature drops to 25–40 °C. In this phase, the most dominant microorganisms are mesophilic microorganisms, including fungi, which are more specialized in degrading starch or cellulose. These mesophilic microorganisms recolonise the compost mix because they either survived the thermophilic phase in the form of spores, or were inoculated in the form of additives, or survived in niches where the temperature was not high enough to kill them. The ecolonization process may be stagnated if the temperature in phase two was higher than 70 °C (Insam & Bertoldi, 2007; Meena et al., 2021). In the last phase, there is an increase in the proportion of fungi relative to bacteria, and there is a formation of lignin-humus complexes which cannot be further degraded (Insam & Bertoldi, 2007). Overall, the process of composting can be summarised as follows (Neugebauer & Sołowiej, 2017):

$$ {\text{Organic waste + microorganisms + O}}_{{2}} \to {\mathrm{H}}_{{2}} {\text{O + CO}}_{{2}} {\text{ + compost + heat}} $$

In the CBP setting, the heating of the material during composting, promotes the evaporation of excess moisture, provided that the pack is periodically cultivated and clumping or matting of the substrate is avoided (Bewley et al., 2017). In CBP systems, new compostable material is added whenever the bedding visually adheres to the animals, and the bedding is turned to mix the manure with the compostable material. This provides a fresh and dry surface for the cows to rest on (Shane et al., 2010). The materials used for CBP must meet the condition of adequate porosity to ensure appropriate aeration during the composting process (Petzen et al., 2009). Research suggests that the temperature rise resulting from microbial activity during composting, controls pathogenic microorganisms (viruses and bacteria) and fly larvae, and inactivates weed seeds, making this an attractive tool for reducing the incidence of mastitis in dairy farms (Eckelkamp et al., 2016b). Other researchers suggest that the management of material mixtures during composting could lead to the proliferation of pathogens responsible for the onset of mastitis, such as coliforms, streptococci, staphylococci, and bacilli (Black et al., 2014; Eckelkamp et al., 2016b). Despite this, no increase in individual or bulk milk tank somatic cell counts or mastitis cases have been observed in CBP systems (Eckelkamp et al., 2016a; Petzen et al., 2009). Bulk milk tank somatic cell count is considered a reliable indicator of herd udder health, prevalence of mastitis within the dairy herd, and milk quality (Constable et al., 2016). Furthermore, CBP can potentially improve cow joint health as concluded in a study by Li et al., (2021).

To date, numerous studies have been published regarding CBP barns, and a recent narrative review has been published, which comprehensively covers the characteristics, health aspects and economic aspects of CBP barns (Leso et al., 2020). Furthermore, a variety of unconventional materials have been used as carbon material sources for composting in this type of system, however, to the authors’ knowledge, there is no review which focuses on the plethora of materials that could be trialled in the CBP setting. The aim of this semi-systematic literature review is to summarise what materials have been tested in this context over the past ten years. Where available, the advantages, and disadvantages that these materials offer will be highlighted. This review will act as a foundation for decision-making when implementing this type of system, by informing which materials can be trialled when CBP barns are being considered by farmers in the United Kingdom. A summary of the factors affecting the composting process will also be provided. The report will have a special focus on alternative materials other than sawdust, wood shavings and woodchips. The latter materials are the industry’s standard (Sobte & Buijs, 2021; Sun et al., 2020). Moreover, because they are valuable materials to produce green energy, they are becoming more expensive and difficult to source (Bjerg & Klaas, 2014)**.** The repurposing of materials that would otherwise end up in the main waste-stream, can be a tool for creating a circular economy for the dairy industry, but would also help dairy farmers to adhere to the following sustainable development goals (SDG): SDG 9 ‘Industry, Innovation and Infrastructure’, SDG 12 ‘Responsible Consumption and Production’, SDG 13 ‘Climate Action’, and SDG 15 ‘Life on land’ (Resolution, 2015).

## Methods

Literature was searched across two databases, Scopus, and Web of Science. The search was restricted to literature published in the last ten years (2014–2023). The keywords for the search were the following: *‘compost barn’, ‘compost dairy’, ‘compost bedded’, ‘compost bedding’, ‘material’.* The first four keywords were selected according to (Silva et al., 2022). To capture literature relevant to the materials that have been used in CBP barns, the search was refined by the addition of the keyword *‘material’*.

The keyword search string including Boolean operators for Web of Science was (TS = (((compost barn*) OR (compost dairy) OR (compost bedded) OR (compost bedding)) AND (material*))) 2014–2023. The keyword search string including Boolean operators for Scopus was (((“compost barn”) OR (“compost dairy”) OR (“compost bedded”) OR (“compost bedding”)) AND (“material*”)) AND PUBYEAR > 2013 AND PUBYEAR < 2024. The difference in the search strings used for the two data bases is owed to the fact that each database has its own search input requirements, therefore the formatting of the search string can vary slightly. The search was conducted on May 22, 2023, therefore, articles published after this date were not included.

A semi-systematic approach was used to select the literature relevant to this report (Snyder, 2019; Wong et al., 2013). The literature was screened by title and abstract to only retain original research papers that specify which alternative material was used in a CBP barn system or composting system. In a subsequent stage, the full text of the remaining literature was screened to assess whether the literature informed about why a certain material was used, and whether there was information about the material’s advantages, disadvantages, benefit to the production system or any other important remarks about the material. During the full text screening stage, the information about materials that is relevant to this report was extracted, with a focus on those papers that included information about alternative materials for CPB barns (alternative to conventional wood shavings, wood chips or sawdust). Additionally, papers known to be relevant to this report, but more than ten years old were also included if they provided information about other materials not covered by the papers obtained from the systematic search. The former studies were found by conducting a manual search. The screening process was completed using the cloud-based solution for systematic reviews Rayyan (Ouzzani et al., 2016). At least two people (screeners) conducted each screening stage (title, abstract and full text), with the blind function turned on so that the screeners could not see other screeners’ decisions. At the end of each stage, the blind was turned off. Once the blind was off, the screeners met to discuss any remaining studies with conflicting decisions and make a final decision on whether the publication would be excluded or included. The retained publications were used to inform the body of this report.

## Results and discussion

The literature search within Scopus and Web of Science returned 193 and 141 papers, respectively. After duplicate removal, 300 remained in total. After the title and abstract screening stage, a total of 94 papers remained, which were divided into a ‘maybe’ category (34) and an ‘included’ category (60). After the full text screening stage, 20 remained in the ‘included’ category. Only open source, original research papers and papers published in English were retained. Papers that only named an alternative material for composting but did not provide any further information about the material itself, or its performance during composting, were excluded. Publications that included at least one alternative material in combination with wood chips, saw dust or wood shavings were retained. The information regarding authors and trialled materials used (whether in regular composting or in a CBP system is presented in Table 1. The information regarding the factors that affect the composting process was obtained from the wider literature, and not strictly from the literature that remained from the screening process.

**Table 1:** Two-way table showing which references discuss a particular material, as indicated by the black squares

	Andrade et al., (2022)	Arango et al., (2023)	Biasato et al., (2019)	Bjerg & Klass, (2014)	Creegan et al., (2022)	Ferraz et al., (2020a)	Ferraz et al., (2020b)	Fávero et al., (2015)	Giambra et al., (2021)	Li et al., (2021)
*Bark mulch*						**■**				
*Barley husk*						**■**				
*Barley straw*					**■**	**■**				
*Biochar*										
*Brushwood*									**■**	
*Cabbage leaves*										
*Carnauba palm*										
*Cereal husks*									**■**	
*Chicken manure*										
*Chopped roots*				**■**					**■**	
*Coffee grounds*										
*Coffee husks*	**■**									
*Coniferous needle litter*						**■**	**■**			
*Corn stover*										
*Crop residue*										
*Distilled grain waste*										
*Domestic food waste compost*			**■**							
*Dried manure*						**■**				
*Flax straw*						**■**	**■**			
*Food waste*										
*Forest biomass*						**■**				
*Garden residuals*				**■**						
*Green sawdust*				**■**		**■**	**■**			
*Heathland vegetation*				**■**						
*Hemp hurd*		**■**								
*Hemp straw*						**■**	**■**			
*Horse manure*		**■**								
*Leaves*				**■**						
*Miscanthus grass*						**■**	**■**			
*Miscanthus mulch*									**■**	
*Peanut shells*								**■**		
*Pecan tree biomass*					**■**					
*Pine tree bark*						**■**				
*Posidonia oceanica*						**■**	**■**			
*Rice straw*										**■**
*Sawdust*	**■**			**■**			**■**		**■**	**■**
*Spelt husks*						**■**	**■**			
*Spent mushroom medium*										
*Swine manure*										
*Triticale husk*						**■**	**■**			
*Triticale straw*							**■**			
*Vegetable waste compost*			**■**							
*Wheat husk*						**■**	**■**			
*What straw*						**■**	**■**			
*Wood chips*				**■**		**■**	**■**		**■**	
*Wood shavings*	**■**						**■**			

	Llonch et al., (2020)	Sampaio et al., (2021)	Sołowiej et al., (2021)	Chung et al., (2022)	Hwang et al., (2020)	Mironov et al., (2021)	Neugebauer and Sołowiej, (2017)	Ravindran et al., (2022)	Wang et al., (2022)	Spiehs et al., (2014)
*Bark mulch*										
*Barley husk*										
*Barley straw*										
*Biochar*				**■**				**■**		
*Brushwood*										
*Cabbage leaves*			**■**							
*Carnauba palm*		**■**								
*Cereal husks*										
*Chicken manure*				**■**	**■**			**■**		
*Chopped roots*										
*Coffee grounds*			**■**							
*Coffee husks*										
*Coniferous needle litter*										
*Corn stover*										**■**
*Crop residue*					**■**					
*Distilled grain waste*									**■**	
*Domestic food waste compost*										
*Dried manure*										
*Flax straw*										
*Food waste*				**■**		**■**	**■**	**■**		
*Forest biomass*	**■**									
*Garden residuals*							**■**			
*Green sawdust*										
*Heathland vegetation*										
*Hemp hurd*										
*Hemp straw*										
*Horse manure*										
*Leaves*										
*Miscanthus grass*										
*Miscanthus mulch*										
*Peanut shells*										
*Pecan tree biomass*										
*Pine tree bark*										
*Posidonia oceanica*										
*Rice straw*										
*Sawdust*	**■**									
*Spelt husks*										
*Spent mushroom medium*					**■**					
*Swine manure*				**■**	**■**					
*Triticale husk*										
*Triticale straw*										
*Vegetable waste compost*										
*Wheat husk*										
*What straw*			**■**			**■**				
*Wood chips*										**■**
*Wood shavings*										

### Factors that affect the composting process

#### Oxygen

Biological oxidation is the main process that makes composting possible. Therefore, the availability of oxygen for the respiration of the aerobic microorganisms is essential. Oxygen also plays a role in the oxidation of the mass’ organic substances. Therefore, it is important to maintain oxygen levels at 18% throughout the process (Bertoldi et al., 1983). Ways of ensuring adequate oxygen levels include blowing, using fans or under-bedding ventilation systems (Giambra et al., 2021; Sampaio et al., 2021), which also help with temperature regulation. High temperatures in the mass are the result of increased metabolic activity of the microorganisms. This augmented metabolic activity will lead to more oxygen consumption, which will result in below-optimal oxygen levels, leading to a decrease in microorganism activity (Finstein et al., 1980).

#### Temperature

Temperatures of between 45 and 55 °C are ideal for microbial activity (Bertoldi et al., 1983). Lower temperatures during the active composting phase will slow the process down, and excessive temperatures (> 70 °C) will inhibit growth of microorganisms. Temperature regulation can be achieved by blowing air at the mass. The blower (via for example forced pressure ventilation or fans) is activated when a ceiling temperature of between 45 and 50 °C is detected through a thermostat, providing a good oxygen supply, and decreasing the temperature at the same time (Finstein et al., 1980). Decreasing temperatures at the last stages of decomposition will encourage the development of actinomycetes and eumycetes, which play a vital role in the decomposition of long-chain polymers, lignin, and cellulose (Bertoldi et al., 1983). The optimal rate of aeration is 0.5–1.0 L/min/kg of compost (Zhang et al., 2019).

#### Moisture

An appropriate level of moisture must also be maintained to ensure that the biological processes involved in composting are not impaired. Moisture levels can be achieved by mixing the organic matter with waste-water sludge (Bertoldi et al., 1983). An optimum moisture level at the start of the composting process, using beef cattle manure and sawdust or rice hull mixes was found to be between 57 and 70% (Kim et al., 2016) which shall then drop to 30% within the first month if composting is carried out appropriately (Bertoldi et al., 1983). However, an optimal range of 50–60% has also been suggested for composting cow manure (Zhang et al., 2019).

#### Carbon to nitrogen ratio

While microorganisms have a carbon-to-nitrogen ratio (C:N ratio) of around 10:1, extensive experimentation has found that the optimal ratio for composting is between 25:1 and 30:1 (Bertoldi et al., 1983; Zhang et al., 2019). Higher ratios will slow decomposition and lower ratios will result in the loss of nitrogen (Bertoldi et al., 1983). The C:N ratio can be corrected by adding manure or sludge to increase the nitrogen content, or by incorporating more organic material (such as sawdust, wood chips, or other carbon material) to boost the carbon content. (Meena et al., 2021).

#### pH

Materials with a pH in the range of 3.0–11.0 can generally be composted, but the optimum range is 5.5–8.0 (Bertoldi et al., 1983). Bacteria thrive in neutral environments while fungi tend to favour slightly acidic conditions. At the beginning of the composting process, the pH typically drops as carbonaceous materials are broken down producing organic acid compounds (Bertoldi et al., 1983).

#### Particle size

In terms of particle size, the smaller the initial size, the greater the relative surface area, therefore increasing the material’s exposure to decomposition and ultimately optimizing the composting process. However, despite this theoretical principle, there is also an ideal particle size (Bertoldi et al., 1983), of less than 25 mm (Shane et al., 2010).

#### Pathogens

Pathogens in compost must be reduced to a minimum to protect public and animal health. During the composting process, saprophytic microorganisms outcompete pathogenic microorganisms (Bertoldi et al., 1983). An example of such competition includes the antibiotic components produced by *Streptomyces spp.* (actinobacteria) (Bertoldi et al., 1983; Insam & Bertoldi, 2007; Kinkel et al., 2012). Other factors such as time–temperature, moisture content, pH, and aeration also have an influence in pathogen inactivation, however, there is no consensus about which conditions the mass must reach to be hygienised, as this will depend on the characteristics of the starting materials, and which pathogens are present in these (e.g., viruses, protozoa, helminths, etc.), and according to a recent review, more research is needing in this area (Lepesteur, 2022). Nevertheless, it is understood that the high temperatures reached during the thermophilic phase, play an essential role in the hygienisation of the composting mix (Meena et al., 2021).

#### Maturity

If the purpose of the compost is growing crops, the composting process must reach the phase of maturity, which is defined as the phase where phytotoxic materials are no longer present (Insam & Bertoldi, 2007). The maturity and stability also determine the quality of the final compost (Goyal et al., 2005). If the compost is used as bedding for livestock, the compost only needs to meet the requirements of recycling of the material, and not of maturity as the compost will not be used for the growing of crops (Zhang et al., 2019). There are several ways in which maturity can be assessed, including enzyme activity tests, assessing the respiratory activity (CO_2_ production or O_2_ consumption), and determining the heat of the compost (Insam & Bertoldi, 2007). Additionally, compost material needs to be devoid of heavy metals and contaminants such as persistent organic pollutants, as these constitute a public health hazard should they enter the food/alimentary chain (Bertoldi et al., 1983; Rigby et al., 2015).

### Materials for composting

#### Fine dry sawdust and wood shavings

Compost dairy barns have been in use in Minnesota since 2001. Barberg et al., (2007b), assessed bedding consisting of fine dry sawdust and wood shavings across 12 dairy farms (Barberg et al., 2007b). The bedding was cultivated and aerated to a depth of approximately 25 cm twice a day. Every two to five weeks, 14 metric tonnes of fresh sawdust were added. However, some farms, did this weekly with lower quantities. During autumn and spring, bedding material was removed from the bedded pack area, and it was cleaned out completely once a year in autumn. Following this, 30–45 cm were added to start a new pack, which by the end of the summer had an average depth of 1.2 m (Barberg et al., 2007b).

When comparing the milk production of the two housing systems (pre-compost and post-compost), at the end of the study, eight of the nine dairies, for which DHIA (Dairy Herd Information Association) data was available, reported a significant increase in milk yield, and nine dairies reported a higher milk fat content (of which three farms had an increase of almost 10%), and a higher protein content (average increase of 3.21%). Furthermore, two thirds of the farms assessed in this aspect recorded a reduction in mastitis incidence, with only one farm recording an increase in this pathology. Additionally, prior to moving the cows to a compost bedded system, mastitis had an overall incidence of 35.4% compared to 27.7% at the end of the study. Reproductive performance and culling were assessed across seven farms. Heat detection increased from 36.9 ± 6.5% to 41.2 ± 7.2% after compost bedding was implemented. Pregnancy rates significantly increased, while annual culling significantly decreased. The incidence of lameness was also low; 7.8% of 793 cows were lame, and two herds had no lame cows at all. The overall satisfaction of the producers was good; however, some expressed concerns about respiratory irritation and eye irritation among the herd following the addition of fresh dry sawdust. In terms of cost–benefit ratios, this bedding system, despite its initial expense, yields greater benefits in reduced culling, improved milk yield and quality, and enhanced reproductive performance, outweighing the additional costs (Barberg et al., 2007b).

#### Wood chips (including leaves, chopped roots, and garden residuals), heathland vegetation, green sawdust, and dry sawdust.

Bjerg and Klaas, (2014) assessed the use of woodchip, heathland vegetation, green sawdust, and dry sawdust in Denmark to establish cheaper and more readily available bedding materials to use in CBP. The trial barn used equipped with an under-floor aeration system to improve water evaporation and reduce the quantity of bedding needed. The aeration system was used hourly for five minutes in winter, and only during the daytime in summer. In this study, a total of four tonnes per cow of material was used, comprising 85% wood chips (including leaves, chopped roots, and garden residuals), 12.5% sawdust and wood shavings, and 2.5% heathland vegetation. Each cow was allocated 10 m^2^, and the bedding depth ranged from 50 to 70 cm, with daily stirring. The investigators estimated that each cow added 7.6 tonnes of manure during the period of December 2012–October 2013. At the end of the study, 3.2 tonnes/cow of compost were removed, while 1.2 tonnes/cow remained in the barn. The authors found that during winter, the water evaporation was insufficient to raise the dry matter content of the bedding. However, in summer, water evaporation proved adequate, eliminating the need for additional bedding material. In this study, ammonia emission was also assessed, and the findings are that the CBP barn system emitted 30% more ammonia than a free stall barn. However, this may be owed to the larger barn size, compared to free stalls (Bjerg & Klaas, 2014).

#### Wood shavings, sawdust, and coffee husks

Andrade et al., (2022) evaluated the use of wood shavings, sawdust and coffee husks during summer and winter in Minas Gerais, Brazil, which is classed as a tropical savanna according to the Kӧppen-Geiger Climate Classification (Beck et al., 2018). The study aimed at assessing, comparing, and characterising the spatial distribution of the main bed variables, and indicators of milk production and animal welfare, in a CBP barn equipped with negative tunnel ventilation. Therefore, the study did not aim to assess bedding material specifically (Andrade et al., 2022).

The CBP material used during the study was a (non-specified ratio) mixture of coffee husks and wood shavings with a depth of approximately 60 cm. However, the starting material was a 30 cm layer of sawdust. The bed was stirred twice a day and new material (sawdust, wood shavings and coffee husks) was added every 12–15 days when the bedding started to adhere to the cow’s skin due to high humidity. A complete change of the bedding material occurred after 12 months, and subsequently every six months. The obtained composted material was used to fertilise an on-farm coffee plantation. The average space per cow in the study was 9.2 m^2^ (Andrade et al., 2022).

The study assessed temperature, humidity, and pH at the surface and a depth of 20 cm. In summer, the surface temperature exceeded the optimal range for dairy cattle comfort (5–25 °C), but it was adequate in winter. Throughout both seasons, mean surface moisture, temperature, and moisture at 20 cm depth were lower than recommended for successful composting. Additionally, pH levels at the surface and 20 cm depth were above the ideal range. The study also revealed that a high animal density per surface area negatively impacted internal bed moisture and temperature, limiting the effectiveness of CBP barns. The C:N ratio was outside the desired range for optimal composting in both seasons (Andrade et al., 2022).

The locomotion and hygiene scores were good during both seasons, with no cow being visibly dirty (score 4) during the study, and only 1.8% of animals showing severe lameness (score 3). Hygiene scores were evaluated according to Schreiner and Ruegg (Andrade et al., 2022; Schreiner & Ruegg, 2002). Furthermore, during the summer 45.6% animals had normal locomotion (score 0) and 45.6% of animals showed mild lameness (score 1). During winter 62.5% of cows showed a lameness score of 1 and 14.8% a score of 0. However, there were signs of discomfort among the cows in summer due to the above-desired temperature (Andrade et al., 2022).

#### Brushwoods, Mischantus mulch, wood chips, cereal husks, broken roots and sawdust

Giambra et al., (2021) carried out a study in Germany where they assessed the presence of thermophilic aerobic sporeformers (TAS) in CBP barns. The study was carried out between December 2017 and February 2019. During winter 2017, the bedding material was composed of wood chips, cereal husks, *Miscanthus* mulch, and sawdust. During winter 2019 broken roots and shredded brushwoods were also included. During the winter of 2019, the moisture content of the bedding was higher (65.62% vs. 50.33%) (Giambra et al., 2021). This could be due to the higher content of broken roots and brushwoods if these were not dried. No further information about the physicochemical characteristics of the materials is provided in the study. However, the authors found that the presence of TAS was negatively correlated with the moisture content of the bedding and the relative humidity of the environment. Furthermore, the presence of TAS was positively correlated to the bedding temperature. The authors explain that this could be due to TAS thriving in higher temperatures, which is only possible in a drier bedding, as higher moisture contents tend to decrease the temperature of the bedding, and hinder aerobic conditions (Giambra et al., 2021). The TAS detected in the bedding during the study period were *Aneurinibacillus thermoaerophilus*, *Bacillus licheniformis*, *Geobacillus thermodinitrificans*, *Laceyella sacchari*, *Thermoactinomyces vulgaris* and *Ueribacillus thermospaericus*. The authors did not study the impacts of these microorganisms on udder health and mentioned that *Geobacillus spp*., and *Bacillus licheniformis* species are considered non-pathogenic. It is also found that some of the TAS detected during the study period, were also detected in cows of the same farm, that were housed in non-CBP cubicles, which likely indicates that those TAS are not correlated with CBP barns. *Thermoactinomyces vulgaris,* for example, was found in stalls with cows that were being fed concentrate, which indicates that the spores of these microorganisms are present in the feed and pass unaffected through the digestive tract of the cow. The concentration of TAS was also not related to the stocking density of the cows in the barn (Giambra et al., 2021).

#### Hemp hurd

Hemp hurd is one of the waste products resulting from the hemp production industry which is increasing in Europe. Hemp hurd has been trialled in Italy as a material for horse bedding mixed with the horse’s manure (Arango et al., 2023). The varieties of hemp (*Cannabis sativa L.*) assessed in the study were Fibranova, USO31, Futura 75, Eletta Campana, Felina 32, Ferimon, CS, Codimono, Carmaleonte and Santhica. Prior to use, the material of ten different varieties of hemp was cut to 60 cm stalks and left to sundry. The mucilage was subsequently removed by rinsing the stalks with water. After a final drying period, the stalks were milled into 8 mm pieces. Although the material was not tested for composting purposes, the researchers found that hemp offers similar water and ammonia absorption capacities compared to wood shavings when used for animal bedding. However, although not statistically significant, the ammonia absorption was widely different among the varieties of hemp tested. The water holding capacities, bulk density, particle size and microbial characteristics were not assessed. Although the authors note that the material needs to be further researched, it was concluded that hemp hurd shows promising properties for use as animal bedding (Arango et al., 2023).

#### Food waste

Biasato et al., (2019) conducted a study in Italy where two groups of Fleckvieh cows were randomly allocated to two different housing systems: a free stall barn (FB) and a CBP barn. Domestic food (60%) and vegetable waste (40%) compost was used as bedding material. The material was distributed in a 50 cm-thick layer, stirred twice a day and no additional material was added. It is worth noting that one author has pointed out a difference between *compost bedding* and *composting the bedding* (Galama et al., 2011). A compost bedding system uses readymade compost that is added as a bedding material to the barn, whereas during bedding composting, the material is being composted while being used as a bedding (Galama et al., 2011). In the study by Biasato et al., (2019) compost bedding was used.

During the 12-month study (January–December), the authors found no difference in body condition, complete blood cell count and clinical chemistry among the cows housed in the two different housing systems. Hygiene and lameness scores were better for the cows in the CBP barn system, and cows housed in this system showed no evidence of hock or hoof lesions. The faecal score (which assess the firmness of the manure) did not present any difference between the two bedding systems. Cows housed in CBP barn presented no macroscopic, clinical, or subclinical mastitis, whereas cows in the free stall system presented subclinical mastitis as per somatic cell count (SCC). SCC was higher in all cows housed in a conventional free stall system. Furthermore, cows housed in CBP barns had fewer agonistic reactions (behavioural evaluation). The milk fat content in CBP-housed cows was higher, however, there was no difference in protein, casein, lactose, or urea. The temperature of the CBP was higher than the environmental temperature, as expected. The authors concluded that animal welfare, milk quality and the quality of milk products (such as cheese) can potentially benefit from this housing system. The bedding’s physicochemical and bacterial analyses were reported. Among the findings of the study were that the pack never reached temperatures (mean 21.02 ± 1.57 °C) high enough to sanitise/hygienise the composting material (Biasato et al., 2019). This could be a consequence of not adding any bulking materials to the mix that have the potential to improve the temperature profile of the mix (Chung et al., 2022; Neugebauer & Sołowiej, 2017; Ravindran et al., 2022). The material temperature was higher than the environmental temperature indicating that there was microbial activity. The authors described this as a semi composting process rather than a composting one (Biasato et al., 2019). The total bacterial count (11.46 ± 0.12 log10 CFU/g of dry matter), Enterobacteriaceae (7.31 ± 0.69 log10 CFU/g of dry matter) and faecal streptococci (5.62 ± 0.48 log10 CFU/g of dry matter) were lower in the CBP than in the FB (control) (Biasato et al., 2019), contrasting with the results of other studies that found lower bacterial counts (Barberg et al., 2007a; Black et al., 2014). Ammonia (312.6 ± 297.0 mg/kg) was also found to be lower in the CBP than in the FB. The studied CBP barn also had a mean pH of 8.72 ± 0.20, agreeing with the values recommended for compost that has reached maturity (Biasato et al., 2019; Maso & Blasi, 2008; Yang et al., 2013). The C:N ratio) was 2.77 ± 0.37 (Biasato et al., 2019), suggesting that the ratio should be improved as it contrasts with the C/N found in other investigations (19.5 (Barberg et al., 2007a), and 26.7 (Black et al., 2014)). Additionally, the salinity was higher in CBP, which may be attributed to the process of composting which released ammonium, mineral salts, and sulphur ions. Finally, the fulvic and humic acids were also higher in the CBP than in FB (Biasato et al., 2019). The amount of these substances is linked to the quality of the final compost (Watteau & Villemin, 2011).

Mironov et al., (2021) conducted a study with the aim of investigating the microbial communities present, and their functions, during the different stages of the composting process. For this study, food and agricultural waste were mixed for composting, yielding high initial humidity percentages and a high initial C:N ratio. The materials were composted in laboratory conditions for 98 days using a bioreactor. Food waste was composed of various vegetables and fruits (including bananas and citrus which have slow composting rates), bread, cheese, fish, and eggs (Mironov et al., 2021; Neugebauer & Sołowiej, 2017). The agricultural waste was wheat straw. Other materials to absorb moisture, such as straw, sawdust, peat, or grain crops, were also included. The proportion of food waste to agricultural waste was 85:15. All materials were fractioned to a size of less than 1 cm. The humidity of the composting mix was maintained at 53–69% by adding tap water, the mass was ventilated mechanically, and the temperature was maintained using heating equipment. After the 98 days had elapsed, the materials were placed on a concrete platform forming a heap and covered with nonwoven material for 10 months. Here the mass was aerated passively. The authors observed that as composting time elapsed, the diversity of microorganisms increased. The transition from the mesophilic stage to the thermophilic stage was characterised by the presence of *Wiesella*, *Leuconostoc* and *Limosilactobacillus*. It was also observed that the highest rates of biodegradation of organic matter occurred during the thermophilic stage were *Caldibacillus*, *Bacillus*, *Aspergillus* and *Penicillium* were the most abundant types of microbiota. The compost output was of acceptable quality for subsequent use in agriculture, and it was concluded that as microbiota diversity increased, so did the properties of plant growth stimulant and development that the compost provided (Mironov et al., 2021).

Neugebauer & Sołovej, (2017), performed a study in which they co-composted food waste (including vegetable and citrus fruit peels, dairy products, and fat) with garden waste as a bulking agent. The proportions of food/household waste to garden waste ranged between 0:1, 1:4, 2:3, 3:2, 4:1, and 1:0, and each mixture was submitted to both a bin and a pit composting method with passive aeration. The latter being where the composting pile is maintained in a pit dug into the ground. The findings were that food waste performed best when composted with at least 40% of garden waste as a bulking agent. The emissions of ammonia were highest when no bulking agent was used, and lowest with garden waste alone or a proportion of 60% garden waste to 40% food waste. Upon finalising the composting process and finding that composting food waste in this manner is viable, the researchers used the output on garden beds and did not observe any adverse effects on the growing plants. However, they did not perform phytotoxicity analyses (Neugebauer & Sołowiej, 2017).

#### Forest biomass (forest cleaning byproduct)

Llonch et al., (2020) compared the use of forest biomass and sawdust in the CBP barn context. The forest biomass consisted mainly of plant fibres and tree bark from a forest in the Mediterranean region. The sawdust that they used had a larger proportion of particles of 2 mm or less compared to the forest biomass. Forest biomass contained more moisture than sawdust, leading to lower temperatures. The C:N ratio was greater for sawdust than for forest biomass bedding. Forest biomass also had a higher apparent density. The material was tilled twice a day, and 800 g/m^2^ (average) were added each day to the bedding if a humidity greater than 60% was detected. The starting depth of the bedding was 30 cm. The study was conducted in Spain from October 2016 to March 2017 and was divided into two 11-week periods separated by a 4-week period in which the cows were placed on traditional wood-shaving bedding. The ambient temperature ranged from 5.9 to 19.5 °C and the humidity from 61.6 to 90.4% throughout the whole study. The results showed that forest biomass was not as appropriate as sawdust for its use in CBP barn based on the physical and chemical properties, but this could be due to the particle size. Bacterial analysis showed that forest biomass could aid in reducing the pathogenic bacterial count in the bedding (Llonch et al., 2020). Therefore, forest biomass could be used as a potential material for CPB barn, but perhaps with a better processing of the material to produce a larger proportion of a particle size closer to 2 mm.

#### Soy hull shavings

Black et al., (2013) assessed CBP barns in Kentucky that used kiln-dried shavings or dry sawdust alone or combined with a mix of soy hull shavings and dry and/or green sawdust. The results showed that after two years of moving the cows into the CBP, milk production increased from 29.3 to 30.7 kg per day, and SCC reduced from 411,230 to 275,510 cells/mL, which may reflect an improvement in milk quality. The SCC was lower than the region’s average (313,000 cells/mL). There was also a reduction in the calving interval. Where only kiln-dried shavings were used, the need for adding bedding material to the CBP was lower, compared to where mixed shavings or green shavings were used (Black et al., 2013).

#### Corn cobs, pine sawdust, pine wood chips and soybean straw

Shane et al., (2010) conducted a study in which pine sawdust was used as a control, against which soybean straw, corn cobs and pine woodchips were compared. The researchers noted that there has been a pressing need for trailing this type of bedding system in seasons other than the Minnesota summer. They were assessed as mixtures at a 2:1 ratio. The mixtures included woodchip fines and sawdust, woodchips and soybean straw, and soybean straw with sawdust, corn cobs alone, sawdust alone, and soybean straw alone (Shane et al., 2010). The study found that these materials and mixtures were all feasible options if the pack was managed appropriately. Corn cob was the driest material, likely due to its smaller particle size, which promotes increased heat. However, the authors noted that the moisture content of the corn cob was below the ideal range (40–65%). The investigators also concluded that regardless of which material or combination is used, it needs to be dry processed to a length of less than 25 mm, offer structural integrity, and a good water holding and absorption capacity (Shane et al., 2010).

#### Rice straw, sawdust and organic manure solids

Li et al., (2021), studied the potential influence of CBP bacterial composition on dairy cow lameness. This study was carried out in China for a period of 50 days. Cows were divided into three groups, one group was housed on conventional sand bedding, the second on concrete floors and the third on CBP (consisting of rice straw, sawdust, and organic manure solids). The relative proportion of materials used and the time of year are not clearly specified. The authors concluded that CBP barns and sand-bedded barns are good alternatives for joint health. They found low abundance of Spirochaetaceae and Treponeme, lameness-associated pathogens, in compost bedding and sand bedding. It suggests that the pathogens linked to digital dermatitis might not be part of the natural microbiota in these bedding systems. Furthermore, there was an improvement in animal welfare and joint health, as indicated by lower concentrations of joint health serum biomarkers (CTX-II and PIIANP) and a better gait performance and hock injury score (Li et al., 2021).

#### Corn stover, particle board, kenaf, peanut hulls and others

Studies to assess the water holding capacities of wood shavings with and without dust, corn stover, kenaf, particle board sawdust, peanut hulls, switchgrass, non-dried sawdust, wheat straw, miscanthus and tobacco stalk compared to kiln dried sawdust (control) were also conducted (Collins, 2011; Fávero et al., 2015). It was concluded that corn stover and kenaf performed similarly to the control. However, all other materials offered less moisture absorption. Non-dried (green) sawdust, peanut hulls, wheat straw, switch grass, and particle board, if intensely managed can perform well (Collins, 2011). Spiehs et al., (2014) compared the performance of corn stover with a variety of wood chips. The authors studied the concentration of odour producing compounds in three types of bedded pack: dried pine wood chips, green and dry cedar chips, and corn stover. These materials were tested in simulation containers for 42 days. The investigators added the bedding material to the containers, and subsequently added bovine urine and faeces to simulate the conditions of a feedlot. The contents of the containers were also stirred in a manner that mimics the presence of cattle walking on the bedding. It was found that green cedar chips produced the highest amount of odour generating compounds such as sulphur compounds, followed by bedding containing dry cedar chips, corn stover and pine chips (in decreasing order) (Spiehs et al., 2014).

Peanut shells have been assessed in a study that evaluated two other farms using saw dust and wood shavings. The aim of the study was to assess the influence of bedding characteristics in the incidence of mastitis, cow cleanliness and bulk milk bacterial populations. The conclusion was that regardless of the material, moisture of the bedding had the highest influence in the development of environmental clinical mastitis, poor cow hygiene and the microbial count in the bulk milk tank. The management of moisture and bedding fluffiness is paramount to improving the hygiene of the animals and reducing the risk of mastitis. The authors recommend using a cow cleanliness scoring to evaluate the risk of subclinical mastitis, and to inform bedding management decisions (Fávero et al., 2015).

#### *Posidonia* oceanica, *Miscanthus* grass, bark mulch and other agricultural waste materials

Ferraz et al., (2020a) assessed the physical properties of 19 different materials across 51 samples. The materials were assessed for air filled porosity, total effective porosity, average particle size, bulk density, global density, container capacity, humidity, saturated humidity, and water holding capacity. Using fuzzy clustering statistical analysis, they clustered the materials into eight groups depending on their physical properties. They found that a good bedding material needs to have a good water holding capacity. Materials with high bulk density, low air-filled porosity and low total effective porosity are unsuitable for CBP barns (Ferraz et al., 2020a). It was concluded that groups three and six which included dried manure, flax straw, fresh sawdust, wood shavings, barley straw, *P. oceanica*, Triticale husk, wheat husk, and wheat straw, were the two most suitable groups of materials for animal bedding. These materials have a saturated humidity which is positively correlated with water holding capacity. The next suitable group of materials was group five which included barley husk, coniferous needle litter, hemp straw, *Miscanthus* grass, and spelt husks. The materials with the worst average values for their physical properties were those in groups seven and eight, which included wheat straw, wood chips, pine tree bark, dry sawdust, and a mix of fresh forest. The reason why some of the above materials are mentioned in different groups is because they were different samples originating from different locations. Therefore, their characteristics could have varied depending on how they were prepared before being assessed, resulting in different physical properties (Ferraz et al., 2020a). The authors also found that water holding capacity had a positive correlation with container capacity, total effective porosity, and saturated humidity, and a negative correlation with air-filled porosity, global density, humidity, bulk density, and average particle size. It was concluded that regardless of the material, it is important to look at the physical properties to decide whether it is suitable for use in animal bedding (Ferraz et al., 2020a).

*P. oceanica* waste is also proposed as an alternative material. In Italy, the waste of this seagrass is a problem during summer season, and it results in a major government expense to remove it from beaches. Because this marine plant showed adequate physical properties in this study, it could be trialled as an alternative material for animal bedding (Ferraz et al., 2020a), within the context of CBP barns. *P. oceanica* is not normally present in the UK, however other seagrass species could be considered, as it is also encouraged by the World Wide Fund for Nature (WWF) (Penciptaan, 2021).

A second study was carried out by the same authors (Ferraz et al., 2020b), in which they analysed 50 samples of 17 materials which they classified as conventional bedding materials (barley straw, dried sawdust, fresh sawdust, triticale straw, wood chips, wood shavings and wheat straw), and alternative bedding materials (barley husks, bark mulch, conifer forest litter, flax straw, hemp straw, *Miscanthus* grass, *P. oceanica*, spelt husks, triticale husks, and wheat husks). The aim of the study was to assess chemical, biological, and physical properties of these materials. The physical properties analysed included water holding capacity, moisture content, bulk density, particle size and dry bulk density. The chemical properties analysed were total organic carbon, nitrogen, and C:N ratio. Finally, the biological properties analysed encompassed count of *Escherichia coli*, *Klebsiella spp.*, coliforms, and total bacterial count. The authors concluded that *P. oceanica* would represent a good alternative bedding material for CBP barns, as well as *Miscanthus* grass and spelt husks, and hemp straw, if the latter is processed to a size of less than 2.5 cm (Ferraz et al., 2020b).

#### Cabbage leaves, wheat straw and coffee grounds

Sołowiej et al., (2021) evaluated the use of cabbage leaves, wheat straw and coffee grounds in various ratios during the composting process using an adiabatic sealed bioreactor equipped with an aeration system. The approximate proportions were the following: pile one contained 100% cabbage leaves, mix two contained 83.3% cabbage leaves and 8.3% wheat straw and 8.3% coffee grounds, and mix three contained 77% cabbage leaves, 8% wheat straw and 15% coffee grounds. The authors concluded that the addition of coffee grounds to approximately 10% into the composting mix had a positive influence on the composting process. The authors noted that the first mix resulted in a C:N ratio of 35:1, while mix two and three had a ratio of 29:1 and 27:1, respectively, which are closer to the ideal ratio for successful composting. This also resulted in a maximum temperature of 80 °C and 75 °C, in mixes two and three respectively, compared to the first mix which only reached 65 °C during the thermophilic phase. Cabbage leaves, wheat straw and coffee grounds individually had a C:N ratio of 20, 125 and 21 respectively (Sołowiej et al., 2021). Although the study was not conducted within and for the context of CBP, but rather, to study the thermophilic phase of the composting process, these could be interesting mixes (mix two or three) to be trialled for potential use in CBP. In the mentioned paper, it is noted that coffee grounds may be somewhat toxic due to the presence of polyphenols, tannins, and caffeine, and that the spent coffee grounds should be processed before composting. It is also noted that if the materials to be composted have a high humidity and low carbon content (such as sewage sludge), additives such as barley or maize straw, or wood chips and sawdust should be added to improve the porosity (Sołowiej et al., 2021). Therefore, it may be important to consider the humidity and carbon content of dairy manure.

#### Biochar

Biochar is a solid which is the resulting product when biomass is submitted to pyrolysis. Pyrolysis is the combustion of biomass (such as woody material) at temperatures exceeding 500 °C without access to oxygen. Biochar is similar to charcoal, with a high porosity, and sorption capacities (Ravindran et al., 2022; Weber & Quicker, 2018), and can be used as a bulking agent to improve aeration and increase the surface area to which microorganisms can adhere to (Ravindran et al., 2022). A study was performed to investigate how rice husk biochar and the addition of salts affects the carbon and nitrogen conservation during the co-composting of food waste and chicken manure. The researchers also evaluated the effect of biochar (and salts) on the final compost output (Ravindran et al., 2022). The mixtures that were tested contained poultry manure, food waste and sawdust at a 2:2:1 ratio, and the first of five mixtures contained these ingredients only. The second mixture consisted of the same components as the first, but with the addition of salts (magnesium hydroxide and potassium hydrogen phosphate), and the third, fourth and fifth mixture contained the addition of 3%, 5% and 10% of biochar respectively. It was found that as the biochar concentration of the mixture increased, the temperature of the composting material also increased. The mixture that contained 10% of biochar reached a temperature of 60 °C (Ravindran et al., 2022), which as stated above, is most ideal for hygienisation of the composting material. Furthermore, water holding capacities had also been improved, and the bacterial growth was enhanced resulting in an improved degradation of the organic matter. The mixtures containing biochar had lower of methane and ammonia. The added salts contributed to the reduced loss of nitrogen. The authors concluded that adding biochar and salts, had a positive effect on the composting process, and aided in the obtention of a better-quality compost, when co-composting chicken manure and food waste (Ravindran et al., 2022). The findings of Chung et al., (2022) were similar. In this instance, the investigators assessed the effects of rice husk biochar when co-composting food waste and livestock manure (chicken and swine) with sawdust. Food waste proportions ranged between 20 and 60%, chicken manure and swine manure between 10 and 30%, sawdust between 15 and 20% and the biochar proportion was either 0%, 3% or 5%, depending on the treatment. The authors found that the bulk density and porosity, which should be ideally low, improved in treatments with biochar. The temperature remained within the optimal temperature range for composting through the addition of biochar, maintaining better microbial activity. The pH was also maintained within desirable ranges for composting in the treatments that included biochar. The same was found with electric conductivity, which is a way of measuring salt contents in the final compost and is related to quality and suitability of compost that will be used in agriculture. Overall, the authors conclude that the amendment of composting material with biochar, improved al physicochemical parameters of the composting material, including the nutritional properties (C:N ratio) of the final product (Chung et al., 2022).

#### Distilled grain waste

Wang et al., (2022) investigated the characteristics of distilled grain waste during composting. The experiment was performed over 65 days in laboratory conditions, in 28-L reactors with an air supply system. The investigators assessed the microbial diversity, its role during the process, and whether there were correlations between microbial communities, composting stages and physicochemical properties of the mixture. At the start of the composting process, the distilled grain waste had a pH of 4.0 which was corrected to 5.0 with the addition of calcium carbonate to ensure a good initiation of the process. The investigators successfully observed all four stages that occur during composting, reaching a mature compost. It was found that distinct microbial communities exist and flourish during the different stages of composting, each with their dedicated metabolic function. These microbial communities were influenced by the physicochemical properties of the composting mass (Wang et al., 2022).

#### Chicken, cattle, and swine manure, spent mushroom medium and crop residue

Hwang et al., (2020), studied the process of co-composting chicken, cattle, and swine manure with crop residue and spent mushroom medium. They assessed the gas emissions and the chemical attributes of the composting material, as well as the link between compost quality and the emission of gases. For the experiment, four different treatments were assessed: chicken manure only, chicken manure with cow manure, chicken manure with plant residue and spent mushroom medium, and chicken manure with swine manure. The humidity of the mixtures was adjusted to 60% through the addition of sawdust. At the end of the composting process, the germination index was evaluated. The germination index provides information about how the compost output performs when used for the germination of plant seeds, and whether the output may be phytotoxic or not. Electroconductivity was also measured, which indicates the presence of soluble salts which can be phytotoxic (Hwang et al., 2020). None of the four treatments showed signs of phytotoxicity. The treatments also reached maturity indicated by a C:N ratio of less than 25. The first stage of the composting process was characterised by an increase in the CO_2_ emissions which dropped to its lowest levels during the maturation phase. The authors attribute the initial increase to the fast degradation of organic matter in the presence of high temperatures. In all treatments, methane emissions were highest during the thermophilic and the mesophilic phases, dropping to almost undetectable levels during maturation. Ammonia emissions were highest during the thermophilic phase, which was attributed to the rapid volatilisation of organic acids. The ammonia emissions declined prior to a second peak, after which they then stabilised at a lower emission level. The authors observed that the treatment containing cow manure, decrease the emission of ammonia. This could have a positive impact on the quality of the final compost, as lower ammonia emissions result in less nitrogen loss, improving the nutrient profile of the final product (Hwang et al., 2020). Another finding was that nitrous oxide was the only gas that did not have an increase in emissions associated to the beginning of the composting process. The pH of the composting pile decreased during and after the thermophilic stage, which was described because of the degradation of acid compounds and a rise in ammonia levels. The authors compared the emissions of the above-mentioned gases among all treatments and concluded that combining chicken manure with cow manure could be a good method to reduce the emission of gases during the composting of chicken manure (Hwang et al., 2020).

#### Suboptimal materials

Coarse hay and cereal grain straw are suboptimal materials due to their tendency of clumping and becoming matted and have therefore been found to not perform well for the purpose of a CBP barn (Bewley et al., 2017). The practice of mixing coconut fibre with wood shavings was attempted by one farmer in a study conducted in Italy (Leso et al., 2013). The result was a rise in moisture content of the pack. Hence, the addition of coconut fibre was inappropriate in this case (Leso et al., 2013). Wet or green sawdust were inappropriate because *Klebsiella spp.* Tend to survive in wood with these characteristics. Using these types of substrates can increase the exposure of the udder to *Klebsiella spp*., which is known to cause environmental mastitis (Janni et al., 2007). However, as mentioned above, green sawdust has been trialled with some success, depending on the management of the CBP and with what other materials it is combined.

Cedar, black walnut and cherry due to being hardwoods, can pose similar problems and predispose horses to laminitis, however it is not known whether the same applies to dairy cows (Bewley et al., 2013).

Pecan tree shreddings (pecan tree biomass) have been trialled for composting together with dairy manure in a windrow system in New Mexico (Creegan et al., 2022). However, pecan trees are deciduous trees and classed as hardwood, therefore, the biomass of these trees may not be safe to use for animal bedding.

A survey conducted among farmers revealed that farmers did not recommend using bean fodder, corn fodder, wheat straw, pine straw, and straw, however the specific reasons are not clear. The main recommendation was to use kiln-dried shavings (Damasceno et al., 2022).

## Conclusion

The literature shows that there is a plethora of materials that can be used for composting, and potentially within the CBP barn system along with dairy cow manure. All materials described above were tested under different settings and in different climates, therefore it is hard to determine whether these materials will be viable in the UK setting. In one of the aforementioned studies there were materials that performed well and others that showed mixed performance depending on how they were processed. It can be concluded that the management and processing of the bedding can change the performance of the material within the CPB barn system. Therefore, the management and processing are perhaps as important, if not more, than the type of material chosen. However, the choice of the material, as was evidenced in this review, can contribute to the quality of the final compost output for its subsequent use in agriculture, and the physicochemical characteristics can be manipulated by altering proportions or amending with bulking agents. Additionally, it is important to consider the origin of the materials chosen, as in some cases these can include persistent organic pollutants or other organic contaminants as remnants from previous industrial processes that the materials underwent. Overall, it can also be concluded that CBP systems can improve animal health, welfare, and farm production. However, it is difficult to establish a clear comparison across materials, as most studies had different focuses and the same parameters were not always evaluated in all studies. Many of the studies cited used materials that most possibly will not be available in the UK, such as Carnauba palm (which grows in Brazil) or pecan tree biomass (which can be expensive and is not grown in the UK). However, other materials could be promising such as particle board leftovers (from the construction industry), *Miscanthus* grass, cabbage leaves, coffee grounds, wheat straw and perhaps even a type of seagrass. These materials are all likely to be readily available in the UK, and it is a matter of liaising with waste management companies to secure a good supply at an acceptable cost, and with the lowest carbon footprint impact possible to ensure a sustainable and clean circular economy. If managed well, CBP barns have the potential of repurposing materials that would otherwise be wasted in the main waste stream. Regardless of which material is chosen for the composting, it is important to consider the particle size with which the compost is started, the C:N ratio, the initial humidity and the water retention capacity and tilling frequency. All these management steps can alter the material’s performance within the CBP barn system. It is also worth mentioning that local legislation should be observed when choosing the materials to be used as a bedding, as some may not be authorised in the effort of preserving animal and public health. Depending on where the materials are sourced, farmers that adopt the CBP system, can potentially alleviate the burdens on local waste management companies, lower the carbon footprint of milk production, increase the sustainability of their production, contribute to the achievement of SDGs, and improve the health and welfare of the cows which are a key working force in the dairy industry.

## Data Availability

The datasets generated and/or analysed during the current study are available from the corresponding author on reasonable request.
